# Effects of Epithelial IL-13Rα2 Expression in Inflammatory Bowel Disease

**DOI:** 10.3389/fimmu.2018.02983

**Published:** 2018-12-18

**Authors:** Bram Verstockt, Clémentine Perrier, Gert De Hertogh, Jonathan Cremer, Brecht Creyns, Gert Van Assche, Marc Ferrante, Jan L. Ceuppens, Séverine Vermeire, Christine Breynaert

**Affiliations:** ^1^KU Leuven, Department of Chronic Diseases, Metabolism and Ageing, Translational Research for Gastrointestinal Disorders, Leuven, Belgium; ^2^University Hospitals Leuven, Department of Gastroenterology and Hepatology, Leuven, Belgium; ^3^KU Leuven, Laboratory of Clinical Immunology, Department of Microbiology and Immunology, Leuven, Belgium; ^4^KU Leuven, Department of Imaging & Pathology, Translational Cell & Tissue Research, Leuven, Belgium

**Keywords:** IL13RA2, goblet cells, anti-TNF non-responsiveness, IBD, inflammatory bowel disease, infliximab, microarray

## Abstract

**Background:** Mucosal IL-13 Receptor alpha 2 (*IL13RA2*) mRNA expression is one of the best predictive markers for primary non-responsiveness to infliximab therapy in patients with inflammatory bowel disease (IBD). The objective of this study was to understand how IL-13Rα2, a negative regulator of IL-13 signaling, can contribute to IBD pathology.

**Methods:** *IL13RA2* knockout (KO) and wild type (WT) mice were exposed to dextran sodium sulfate (DSS) in drinking water to induce colitis. Furthermore, mucosal biopsies and resection specimen of healthy individuals and IBD patients before the start of anti-tumor necrosis factor (anti-TNF) therapy were obtained for immunohistochemistry and gene expression analysis.

**Results:** After induction of DSS colitis, *IL13RA2* KO mice had similar disease severity, but recovered more rapidly than WT animals. Goblet cell numbers and mucosal architecture were also more rapidly restored in *IL13RA2* KO mice. In mucosal biopsies of active IBD patients, immunohistochemistry revealed that IL-13Rα2 protein was highly expressed in epithelial cells, while expression was restricted to goblet cells in healthy controls. Mucosal *IL13RA2* mRNA negatively correlated with mRNA of several goblet cell-specific and barrier genes, and with goblet cell numbers.

**Conclusions:** The data suggest that IL-13Rα2 on epithelial cells contributes to IBD pathology by negatively influencing goblet cell recovery, goblet cell function and epithelial restoration after injury. Therefore, blocking IL-13Rα2 could be a promising target for restoration of the epithelial barrier in IBD.

## Introduction

Crohn's disease (CD) and ulcerative colitis (UC) are chronic inflammatory disorders of the gastrointestinal tract with a relapsing and remitting course. They are thought to result from an excessive immune response toward the intestinal and colonic microbiota in genetically susceptible hosts (1). Current treatments, able to achieve and maintain clinical remission and mucosal healing, include anti-tumor necrosis factor (anti-TNF) agents. Despite an overall good response to anti-TNF therapies, 10–30% of patients are primary non-responders (2). Among several other genes, our group previously identified *interleukin 13 receptor alpha 2* (*IL13RA2)* mRNA expression as one of the best predictive markers for anti-TNF non-responsiveness. As this holds true both in UC and in CD, two diseases mediated by different combinations of cytokines (1, 3, 4), these results point toward a role of IL-13Rα2 in a mechanism common to both diseases. In a more recent study we also found that *IL13R*A*2* expression reflects an increased TNF burden in non-responders (5). However, how IL-13Rα2 affects the response to anti-TNF therapy is currently unknown.

The IL-13 signaling receptor complex consists of two chains (IL-13Rα1 and IL-4Rα) which, after ligand binding, activate the transcription factor STAT-6 (6). IL-13Rα2 is a transmembrane receptor which binds IL-13 with very high affinity. Due to its very short cytoplasmic region lacking signaling motifs, IL-13Rα2 is generally thought to act primarily as a “decoy” receptor, sequestering IL-13 and thus preventing excessive IL-13 signaling via the complex IL-13Rα1 and IL-4Rα (7). In rodents, IL-13Rα2 is released as a soluble factor and its expression correlates with a decreased responsiveness to IL-13 in chronic granulomatous inflammation in the liver (8), airway inflammation and airway-hyperreactivity (9), and immune and functional response to nematode infection (10). Humans do not alternatively splice the *IL13R*α*2* transcript and exclusively express this receptor as a transmembrane molecule (6, 11). However, it has been shown that also in humans, membrane IL-13Rα2 reduces responsiveness to IL-13 (12–14).

The present study aimed to identify how *IL13RA2* expression can contribute to the pathology of IBD. First, we performed experimental studies with *IL13RA2* KO and WT mice subjected to experimental colitis. Next, we determined the localization of the IL-13Rα2 protein in human colonic biopsies by immunohistochemistry. Based on the murine findings and because it is known that IL-13 influences goblet cells, we further correlated *IL13RA2* expression with goblet cell specific genes, barrier genes and goblet cell numbers in IBD.

## Materials and Methods

### Animals and Experimental Colitis Model

*IL13RA2* knock out (KO) mice backcrossed more than 10 times in the Balb/c background were obtained from Pfizer (New York, NY, USA). Both *IL13RA2* KO and wild type (WT) Balb/c mice were bred in the specific pathogen free area of the animal care facility of the KU Leuven, Belgium. Acute colitis was induced by administration of dextran sodium sulfate (DSS, MP Biomedicals) in drinking water (3%) for 7 days, followed by a recovery period. Mice were sacrificed by injection of pentobarbital at day 9 or 12 for evaluation of markers of disease activity and inflammation. Colons were harvested, weighted and measured from the ileocecal junction to the anus. A macroscopic score of inflammation was given as previously described (15). One part of the most infiltrated distal colon was fixed in 6% paraformaldehyde for 3 h and then embedded in paraffin for histology evaluation.

### Evaluation of Inflammation in Mice by Histology

Five μm-thick longitudinal and transverse sections of colons were stained with hematoxylin and eosin (H&E). Three sections per mouse were blindly and independently scored by a post-doctoral researcher (CP) and by an experienced IBD pathologist (GDH). The following five characteristics were scored cumulatively. Architectural disturbance, based on irregularity of the mucosal surface, crypt distortion and branching, loss of glands, and fraying of the muscularis mucosae (0: none, 1: focal and mild, 2: multifocal, or diffuse, 3: severe); goblet cell depletion (0: normal, 1: focal, 2: multifocal, and 3: generalized); epithelial defects (0: none, 1: focal erosion, 2: multifocal erosion, and 3: ulceration); neutrophil infiltration (0: none, 1: in the lamina propria, 2: in the lamina propria with crypt abscesses, 3: infiltration in the mucosa or in the submucosa, or in the muscularis propria or in the subserosa, to a maximum of 12 if several areas were infiltrated); mononuclear cell infiltration (0: within normal limits, 1: slightly increased in lamina propria, 2: dense infiltrate in the lamina propria, 3: cell aggregates in the mucosa or in the submucosa or in the muscularis propria or in the subserosa, to a maximum of 12 if several areas were infiltrated).

### Immunohistochemistry for Human IL-13Rα2 and IL-13Rα1 and Quantification of Goblet Cells in Mucosal Biopsies of IBD Patients

Immunohistochemical staining was performed on 5 μm-thick step sections prepared from paraffin formalin-fixed endoscopic-derived mucosal biopsies and resection specimens from IBD patients and controls. Endogenous peroxidase activity was blocked in deparrafined sections by incubating the slides for 20 min in a 0.3% solution of H_2_O_2_ in methanol. Epitope retrieval was performed by heating the slides for 30 min in Tris/EDTA buffer (pH 9) at 98°C. Sections were then incubated with the anti-human IL-13Rα2 mouse monoclonal antibody clone ab55275 (Abcam plc, Cambridge, UK) at a concentration of 1 μg/ml for 30 min. IL-13Rα1 protein was localized using anti-human IL-13Rα1 rabbit polyclonal antibody ab79277 (Abcam) at a concentration of 10 μg/ml. The Dako REALTM EnvisionTM Detection System kit (Dako Belgium NV, Heverlee, Belgium) was used for visualization of bound primary antibody according to the manufacturer's instructions. Formalin-fixed, paraffin-embedded surgical biopsies of an ovarian serous adenocarcinoma served as positive controls for IL-13Rα2 (16). The primary antibody was omitted in the negative controls.

In parallel, H&E stained sections of mucosal biopsies were used to quantify goblet cells. Numbers of goblet cell were counted manually on images of the biopsies. Each picture covers a total surface of 0.32 mm^2^. The total length of the epithelium on the picture was measured using ImageJ, and data were expressed as number of goblet cells per μm of epithelium.

### Gene Expression Study in Mucosal Biopsies of IBD Patients

Publically available microarray data (Affymetrix Human Genome U133 Plus 2.0 Arrays, GSE14580, GSE12251, and GSE16879) of inflamed mucosal biopsies of IBD patients prior to their first infliximab administration were analyzed. Patient characteristics at baseline were previously described (17, 18). Raw data were preprocessed {normalization robust multichip average (RMA) metho(19)}, , and quality-control evaluation was performed [arrayQualityMetrics (20)] using Bioconductor (http://www.bioconductor.org) in R version 3.5.0 (R Development Core Team, Vienna, Austria), whereafter outliers were detected and removed based on cluster analysis (21). Microarray data were used to quantify mRNA expression of goblet cell specific and barrier genes.

### Statistical Analysis

Murine data analysis was performed with GraphPad Prism 5 (La Jolla, CA, USA). Data are represented as medians, and *P*-values were obtained using two-tailed Mann-Whitney *U* testing. Differences were considered statistically significant at *p* < 0.05. Human data analysis was performed in R (R Development Core Team, Vienna, Austria). Correlations between *IL13RA2* expression and goblet cell related genes and numbers were analyzed using bivariate two-tailed correlation tests.

## Results

### *IL13Rα2* KO Mice Are Equally Affected in Acute DSS Colitis as WT Mice, but Recover Faster

**Figure 1:**
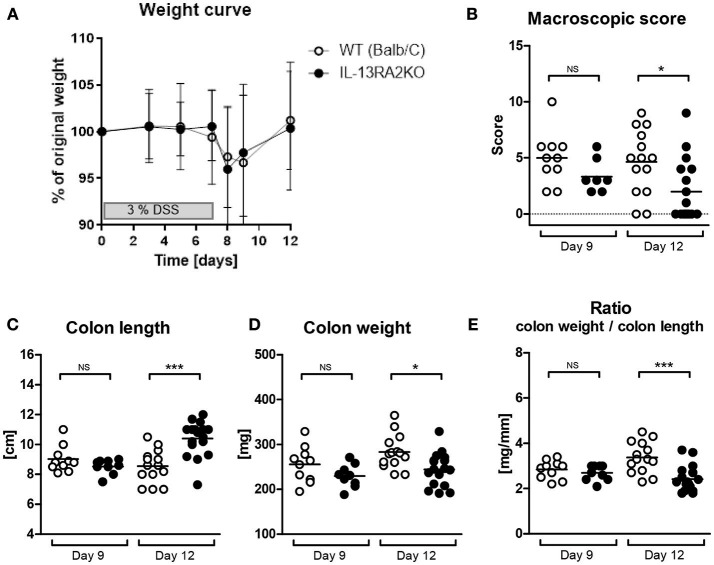
Acute colitis in WT and *IL13RA2* KO mice. Wild type (WT) and *IL13RA2* knock out (KO) mice were exposed for 7 days to 3% DSS in drinking water to induce acute colitis. Data are pooled from two independent experiments including 24 WT mice (day 9: *n* = 10 and day 12: *n* = 14) and 26 *IL13RA2* KO mice (day 9: *n* = 9 and day 12: *n* = 17). WT mice are shown in white, *IL13RA2* KO mice in black. Weight curve **(A)**; Macroscopic score of inflammation of the colon at day 9 and day 12 after start of DSS exposure **(B)**; Length of colon **(C)**; Weight of colon **(D)**; Ratio colon weight on length **(E)**. Data are represented as medians (^*^p < 0.05, ^***^p ≤ 0.001, NS, not significant).

Acute colitis was induced in *IL13RA2* KO and WT mice by DSS administration. At day 9, weight loss was most severe in both strains, reflecting the time point of strongest inflammation. At day 12 (recovery phase), most mice had recovered their initial weight despite persistent inflammatory features in the colon. Both strains developed colitis and equally lost weight (*p* = 0.07 at both day 9 and 12) (Figure 1A). The inflammation of the colon, as evaluated by the macroscopic score, the colon length, the colon weight and the colon weight to length ratio, was similar in *IL13RA2* KO and WT mice at day 9 (*p* = 0.10, 0.34, 0.18, and 0.41, respectively) (Figures 1B–E). In contrast, at day 12, colons of *IL13RA2* KO mice were less inflamed than colons of WT mice, with significant differences in the macroscopic score of inflammation (*p* = 0.01), the colon length (*p* < 0.001) and colon weight (*p* = 0.01), reflecting that inflammation resolved more rapidly in *IL13RA2* KO mice.

**Figure 2:**
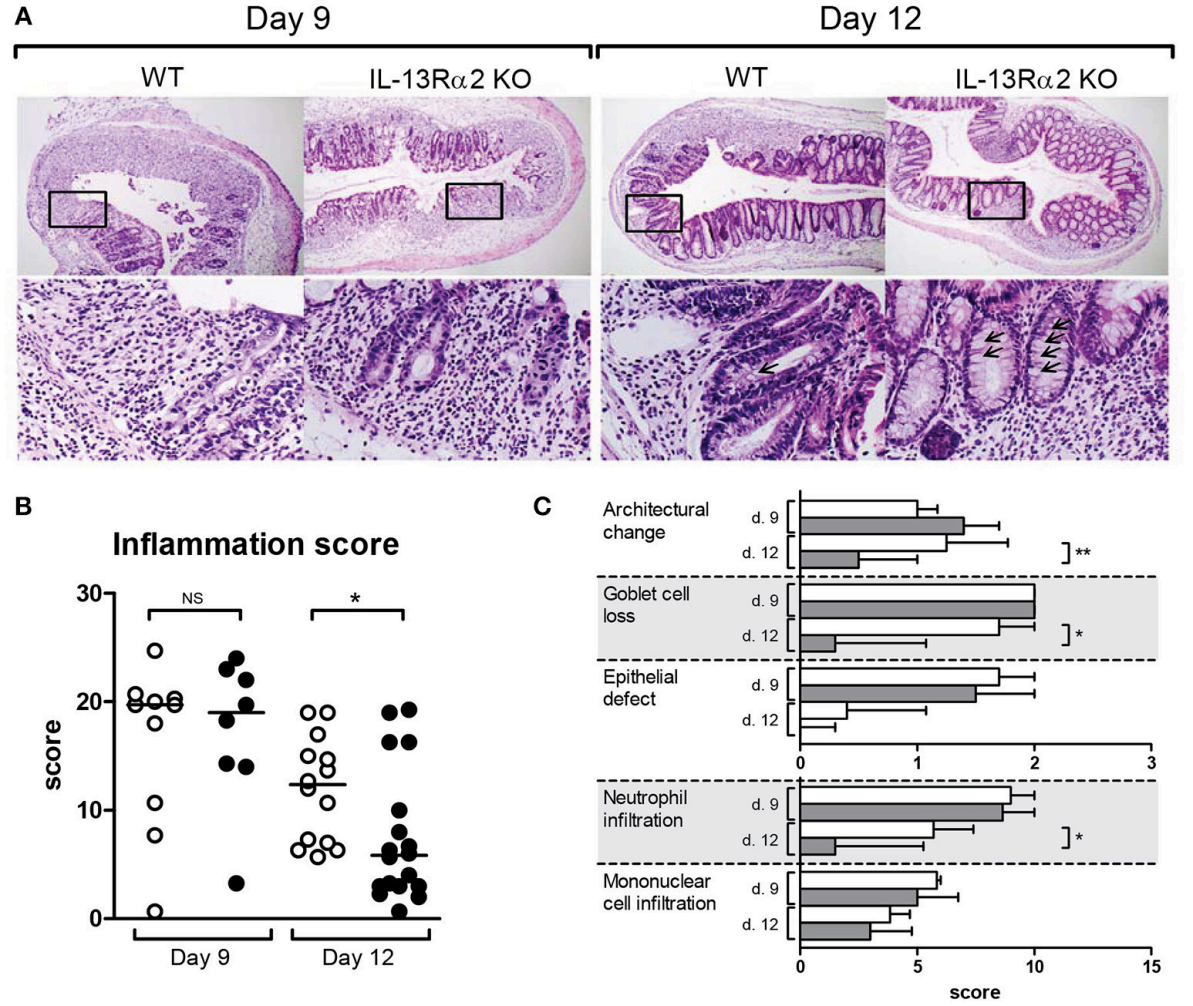
Microscopic score of inflammation in mice with DSS colitis. Wild type (WT) and *IL13RA2* knock out (KO) mice were exposed for 7 days to 3% DSS in drinking water to induce acute colitis. Data are pooled from two independent experiments including 24 WT mice (day 9: *n* = 10 and day 12: *n* = 14) and 26 *IL13RA2* KO mice (day 9: *n* = 9 and day 12: *n* = 17). WT mice are shown in white, *IL13RA2* KO mice in black. Representative pictures of haemotoxylin and eosin (HandE) stained cross sections of colon of mice. The lower pictures represent the area in the rectangle at higher magnification **(A)**; Inflammatory score evaluated on histological sections **(B)**; Details of the inflammatory score **(C)**. Data are represented as medians (^*^p < 0.05, ^**^p ≤ 0.01, NS, not significant).

Inflammation was microscopically quantified on H&E stained colon sections (Figure 2A). Microscopically, the score of inflammation was not different between *IL13RA2* KO and WT mice at day 9 (*p* = 0.82), but it was significantly lower in *IL13RA2* KO mice at day 12 (*p* = 0.02) (Figure 2B). At day 12, more goblet cells were present in the epithelium of *IL13RA2* KO mice (arrows). More specifically, the characteristics that were different between the two strains of mice on day 12 were the architectural changes (*p* = 0.009), the goblet cell depletion (*p* = 0.01) and the infiltration of neutrophils (*p* = 0.03) (Figure 2C). Taken together, these data demonstrate that the absence of IL-13Rα2 allows a more rapid recovery of the mucosa and in particular for the regeneration of goblet cells and normalization of the architecture of the colonic mucosa. IL-13Rα2 thus seems to negatively regulate epithelial/mucosal healing.

### IL-13Rα2 Is Expressed in Human Epithelial Cells

**Figure 3:**
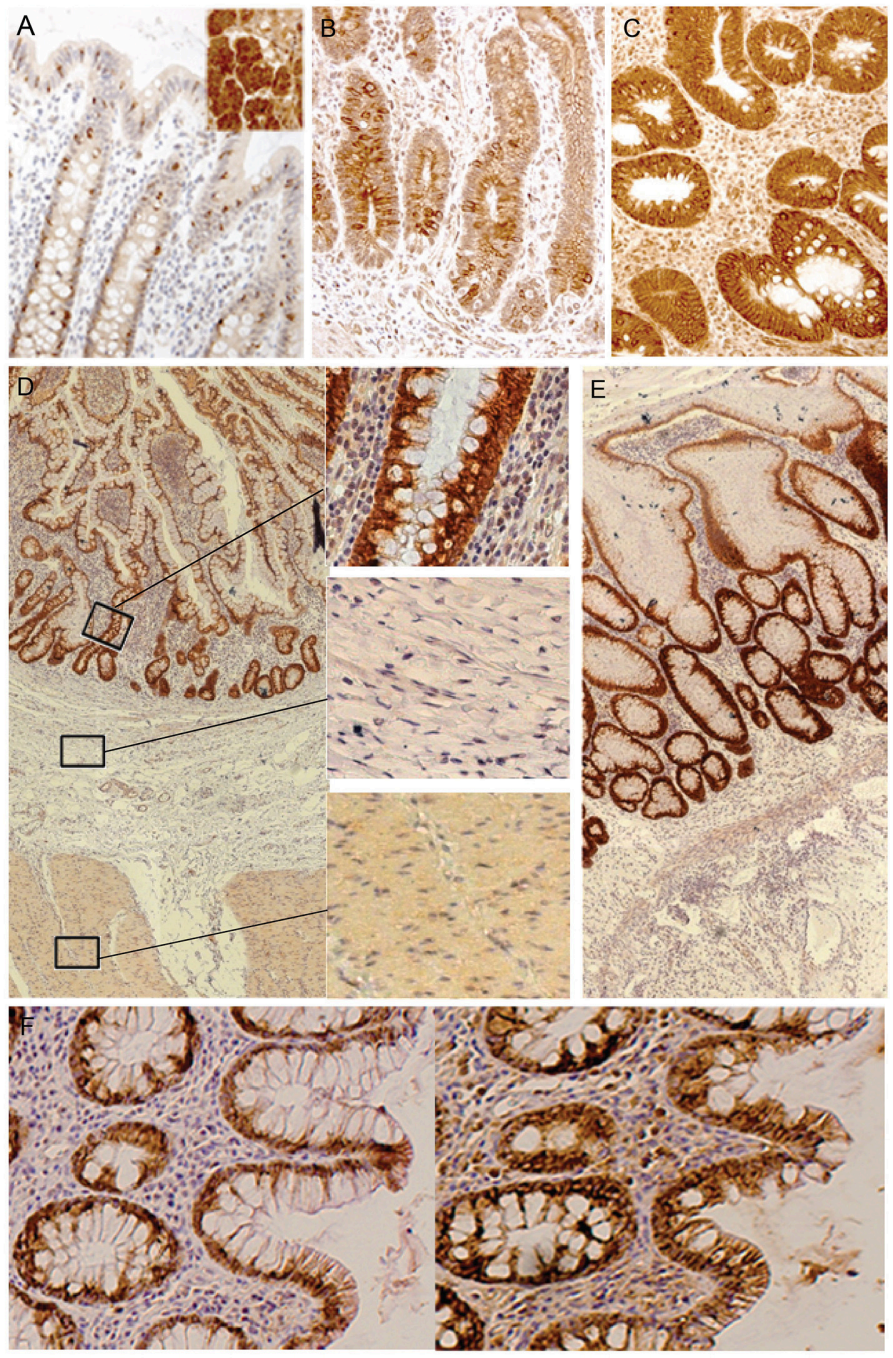
Immunohistochemical detection of IL-13Rα2 and IL-13Rα1 in human tissues. Staining of IL-13Rα2 protein with specific antibody in human tissues: normal colon and in the insert an ovarian serous adenocarcinoma as positive control (16) (negative controls where the first antibody was omitted are not shown) **(A)**; Ileum of a CD non-responder before therapy **(B)**; Colon of an UC non-responder before therapy **(C)**; Resection specimen of CD **(D)**; Resection specimen of UC **(E)**; Sequential staining of colon of UC non-responders for IL-13Rα2 (left) and IL-13Rα1 (right) **(F)**. The black bar corresponds to 100 μm.

Using mucosal gene expression gene signaling analysis, we previously reported that *IL13RA2* is highly expressed in the mucosa of IBD patients, and more so in anti-TNF non-responders (5, 17, 18). We therefore determined the cellular localization of the IL-13Rα2 protein by immunohistochemistry on mucosal surface biopsies and full thickness resection specimen of IBD patients and healthy controls. In normal colon, IL-13Rα2 was localized in the cytoplasm and on the membrane of the goblet cells (Figure 3A). In inflamed CD and UC biopsies, there was increased staining intensity for IL-13Rα2 in all epithelial cells (Figures 3B,C). The staining was very intense at the base of the crypts and became slightly less intense in cells at the tip of the villi. We did not observe any staining in lymphoid aggregates, nor in cells surrounding fibrotic areas in resection specimen of CD and UC patients (Figures 3D,E). Immune cells, such as macrophages and monocytes, as well as fibroblasts did not express IL13RA2, even in the deeper layer of the tissues. In resection specimens of patients with colorectal carcinoma, IL13RA2 was also expressed in epithelial cells surrounding the tumor, although the intensity of the staining was slightly weaker than in CD and UC patients. The IL13RA2 expression in these patients suggests that it is not specific to IBD, but might be a consequence of chronic inflammation.

As IL-13 signals via the complex IL-13Rα1/IL-4Rα, we analyzed whether IL-13Rα1 was also expressed in epithelial cells of IBD patients. In patients with strong inflammation, IL-13Rα1 was co-expressed in epithelial cells together with IL-13Rα2 (Figure 3F). As expected, IL-13Rα1 was also expressed in lymphoid cells in the lamina propria (data not shown).

### Expression of *IL13RA2* mRNA Negatively Correlates With Expression of Goblet Cell Related Genes and Goblet Cell Numbers in the Mucosa of IBD Patients

**Figure 4:**
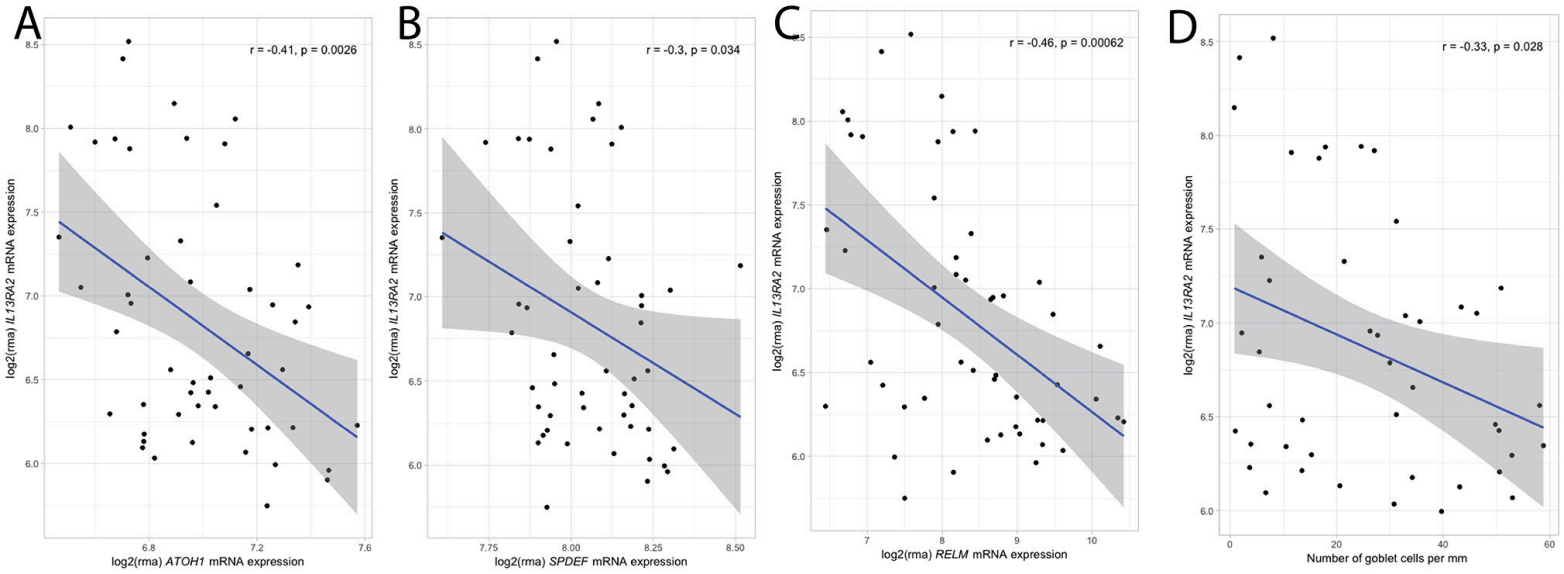
Correlation between *IL13RA2* mRNA and mRNA of goblet cell related genes. Correlations between expression of *IL13RA2* with the expression of *ATOH1* **(A)**, *SPDEF* **(B)**, *RELM*β **(C)** in mucosal biopsies of IBD patients before anti-TNF therapy. Correlations between expression of *IL13RA2* with the number of goblet cells per mm in IBD patients prior to start of anti-TNF therapy **(D)**.

In follow up of our murine findings on goblet cell recovery in the absence of IL-13Rα2, we analyzed the correlation between *IL13RA2* expression and the expression of various goblet cell related genes. Mucosal expression of *ATOH1*, a transcription factor specific for the development of goblet cells, *SPDEF*, a key promotor of goblet cell differentiation and regulator of secretory gene products including mucin 2, and *RELM*β, a key goblet cell mediator, were negatively correlated with *IL13RA2* expression (Figures 4A–C). Additionally, *IL13RA2* mRNA expression correlated inversely with gene expression of key components of the intestinal mucus layer (22), including *FCGBP and CLCA1* (*r* = −0.5, *p* = 4.0 × 10^−4^; *r* = −0.4, *p* = 2.0 × 10^−3^, respectively) (Supplementary Figures 1A,B). To strengthen these findings, the number of goblet cells per mm epithelium was quantified in mucosal biopsies of IBD patients, and correlated negatively with the expression of *IL13RA2* (*r* = −0.33, *p* = 0.03) (Figure 4D). Thus, the expression of *IL13RA2* prior to therapy is associated with lower numbers of goblet cells, lower expression of transcription factors for goblet cell development and goblet cell function. This suggests that IL-13Rα2 negatively regulates development of goblet cells during inflammation, most likely by counteracting the effects of IL-13.

In general, these murine and human findings suggest a link between IL13RA2 and mucosal barrier function. Based on previous findings demonstrating an intestinal epithelial barrier dysfunction in IBD, with up- (*CDN1, DSG3*) or downregulation (*CLDN8, OCLDN, MEP1A*, and *MAGI1*) of particular genes in active IBD compared to healthy individuals (23), we studied the correlation between *IL13RA2* expression and these barrier genes. Although causality cannot be proven, we found a significant positive correlation between *IL13RA2* and *CDN1* (*r* = 0.38, *p* = 0.006) and *DSG3* expression (*r* = 0.61, *p* = 2.2 × 10^−6^), whereas *IL13RA2* correlated negatively with *CLDN8* (*r* = −0.38, *p* = 0.006), *OCLDN* (*r* = −0.44, *p* = 0.001), *MEP1A* (*r* = −0.35, *p* = 0.01), *MAGI1* (*r* = −0.43, *p* = 0.002), and *MARVELD2* (*r* = −0.32, *p* = 0.02).

## Discussion

Previously, *IL13RA2* mRNA expression has been identified as one of the best predictive markers for anti-TNF non-responsiveness (5). However, how IL-13Rα2 interferes with the effect of anti-TNF therapy is currently unknown. The present study aimed to unravel the effects of mucosal IL-13Rα2 expression in IBD. We first hypothesized that IL-13Rα2 downregulates the inflammatory process, as it has been shown to inhibit several IL-13-mediated effects in mice (8–10, 24). IL-17A/IFNγ-mediated colitis in *IL10* KO mice is regulated indirectly by soluble IL-13Rα2 (25), which blocks the inhibitory function of IL-13 on Th17 cells, and thus promotes Th17 activities (26). However, we did not notice any difference in macro- or microscopic inflammation between WT and *IL13RA2* KO mice in this acute DSS colitis model, arguing against a role for IL13RA2 in the inflammatory process. Still it might be important to study other models of IBD. The DSS model was chosen for our study because DSS triggers inflammation by disruption of the epithelial barrier (27), and is as such an appropriate model to study mucosal healing. Importantly, using this model we indeed did observe a more rapid epithelial recovery in *IL13RA2* KO mice, including more goblet cells at day 12. These findings suggest that *IL13RA2* expression is not influencing susceptibility to colitis, but instead influences the recovery after removal of the trigger in mice, and that in particular effects on goblet cells are important. Indeed, also in human genome-wide association studies in IBD already pointed toward distinct mechanisms between disease susceptibility and disease outcome, once the disease has settled (28).

Besides its effects on inflammation, IL-13 has been shown to promote airway epithelial cell proliferation (29–31), and to prevent apoptosis (32), features that are crucial for epithelial cell recovery. IL-13 has an established role in the differentiation of goblet cells in the airways (30, 33, 34), and overexpression of IL-13 in the gut results in villus blunting, goblet cell hyperplasia and increased epithelial cell proliferation (35). The role of IL-13 in goblet cell hyperplasia is further supported by studies showing that stimulation of primary lung epithelial cells by IL-13 causes an increase in the population of goblet cells (33). These effects are likely to be mediated via IL-13Rα1 and STAT6. Co-expressed IL-13Rα2 can act as a negative regulator of IL-13 effects, reducing signaling via IL-13Rα1, and thus reducing the above-mentioned beneficial effects of IL-13 in the gut. It has already been demonstrated that *IL13RA2* expression is inversely correlated with STAT-6 phosphorylation (36–38). As STAT-6 is key in goblet cell hyperplasia and intestinal mucosal repair (39, 40), the observed goblet cell hyperplasia in IL13R*A*2 KO mice can easily be explained.

Interestingly, when we studied biopsies of patients with IBD, we confirmed an inverse correlation between *IL13RA2* expression and total number of goblet cells, as well as between *IL13RA2* expression and the expression of several goblet cell specific and barrier genes. We therefore hypothesize that the increased *IL13RA2* expression dampens one's ability to restore the mucosal barrier and hence impairs mucosal healing. Although goblet cell hyperplasia and mucus production contribute to inflammation in asthma (41), these mechanisms are protective in the gut (42, 43). Translated to IBD patients this may explain why some patients achieve mucosal healing and others do not, after receiving the same anti-TNF agent, neutralizing one of the driving forces and upstream regulators of *IL13RA2* expression (5). Additionally, blocking IL-13Rα2 could be a promising agent for restoration of the epithelial barrier in IBD.

We cannot exclude that IL-13Rα2 also influences disease by other mechanisms. Besides IL-13, another ligand for IL-13Rα2, chitinase 3-like 1 (CHI3L1/YKL-40) in complex with TMEM219 (the chitosome) or Galectin-3 (Gal-3), can regulate a variety of cellular and tissue responses (14, 44, 45). CHI3L1, secreted by macrophages and neutrophils, plays essential roles in the pathogen clearance and generation of the host tolerance (46). The CHI3L1-IL13RA2 axis is known to play a critical role in cell death, inflammasome activation, Th1/Th2 cytokine balance, and Erk, Akt, and Wnt/β-catenin signaling (45). Recent data suggested that IL-13Rα2 is an α-receptor subunit that can interact with TMEM219 to mediate anti-apoptotic effector responses, whereas IL-13Rα2 interactions with Gal-3 would augment apoptotic responses and Wnt/β-catenin activation (45). Additional research is necessary to further unravel the significance of these effects of IL-13RA2, also in IBD.

In conclusion, *IL13RA2* expression correlates negatively with numbers of goblet cells, as well as with the expression of goblet cell transcription factors and goblet cell function, suggesting that high *IL13RA2* expression impairs goblet cell development. The role of IL-13Rα2 is further supported by murine data showing a more rapid recovery of *IL13RA2* KO mice compared to WT mice after DSS induced colitis, with a more rapid restoration of goblet cells. In anti-TNF therapy non-responder patients in whom there is increased *IL13RA2* expression (5, 17, 18), the latter probably hampers the renewal of the epithelium and the differentiation of goblet cells that would otherwise be induced by IL-13 via the IL-13Rα1/IL-4Rα complex. Neutralization of TNF by anti-TNF therapies decreases the inflammation via several mechanisms (47), but it is conceivable that this can be efficient only if the epithelium recovers properly to prevent entry of commensal bacteria and/or bacterial products contributing to the inflammation.

## Ethics Statement

All patients included in the analysis had given written consent to participate in the Institutional Review Board approved IBD Biobank (B322201213950/S53684), collecting serum, biopsies and clinical characteristics among other items. All murine studies were approved by the local ethical committee for animal experimentation of the KU Leuven(P013/2011).

## Author Contributions

BV: contributed to the acquisition of data, analysis and interpretation of data, drafting of the manuscript, and statistical analysis. CP: study concept and design, acquisition data, analysis and interpretation of data, and drafting of the manuscript. GDH: acquisition data, analysis and interpretation of data, and critical revision of the manuscript for important intellectual content. JC: acquisition data, analysis, and interpretation of data. BC: interpretation of data. GVA: study concept and design, interpretation of data, and critical revision of the manuscript for important intellectual content. MF and SV: acquisition of the data, interpretation of data, and critical revision of the manuscript for important intellectual content. JLC: study concept and design, interpretation of data, and critical revision of the manuscript for important intellectual content. CB: study concept and design, acquisition of data, analysis and interpretation of data, material support, drafting of the manuscript, and study supervision. All authors agreed with the final version of the manuscript. CB: guarantor of the manuscript.

### Conflict of Interest Statement

BV received financial support for research from Pfizer; lectures fees from Abbvie, Ferring, Takeda Pharmaceuticals, Janssen and R Biopharm; consultancy fees from Janssen. GDH received consultancy fees from Centocor and Takeda. GVA received financial support for research from Abbott and Ferring Pharmaceuticals; lecture fees from Janssen, MSD and Abbott; consultancy fees from PDL BioPharma, UCB Pharma, Sanofi-Aventis, Abbott, Abbvie, Ferring, Novartis, Biogen Idec, Janssen Biologics, NovoNordisk, Zealand Pharma A/S, Millenium/Takeda, Shire, Novartis, and Bristol Mayer Squibb. MF received financial support for research from Takeda and Janssen; lecture fees from Ferring, Boehringer- Ingelheim, Chiesi, Merck Sharpe andamp; Dohme, Tillotts, Janssen Biologics, AbbvieTakeda, Mitsubishi Tanabe, Zeria; consultancy fees from Abbvie, Boehringer-Ingelheim, Ferring, Merck Sharpe andamp; Dohme, and Janssen Biologics. SV received financial support for research from MSD, Abbvie, Janssen and UCB Pharma; lecture fees from Abbott, Abbvie, Merck Sharpe andamp; Dohme, Ferring Pharmaceuticals and UCB Pharma; consultancy fees from Pfizer, Ferring Pharmaceuticals, Shire Pharmaceuticals Group, Merck Sharpe andamp; Dohme, and AstraZeneca Pharmaceuticals. The remaining authors declare that the research was conducted in the absence of any commercial or financial relationships that could be construed as a potential conflict of interest.

## Supplementary Material

Supplementary Figure 1 Correlation between *IL13RA2* mRNA and goblet specific cell genes. Correlations between expression of *IL13RA2* with the expression of *FCGBP* **(A)** and *CLCA1* **(B)**, backbone of the colonic mucus layer.

## Abbreviations

ATOH1: atonal BHLH transcription factor 1
CD: Crohn's disease
CHI3L1: chitinase 3 like 1
CLCA1: chloride channel accessory 1
DSS: dextran sodium sulfate
FCGBP: FC fragment of IgG binding protein
GAL3: galectin 3
H&E: haemotoxylin and eosin
IBD: inflammatory bowel disease
IL: interleukin
KO: knock out
RELMβ: resistin like beta
SPDEF: SAM pointed domain containing ETS transcription factor
TMEM219: transmembrane protein 219
TNF: tumor necrosis factor
UC: ulcerative colitis
WT: wild type
YKL-40: chitinase 3 like 1.
