# Supplementary figures and images for: Effects of Epithelial IL-13Rα2 Expression in Inflammatory Bowel Disease
Source: Front Immunol. 2018 Dec 18;9:2983. doi: 10.3389/fimmu.2018.02983 (PMC6305625; doi:10.3389/fimmu.2018.02983)

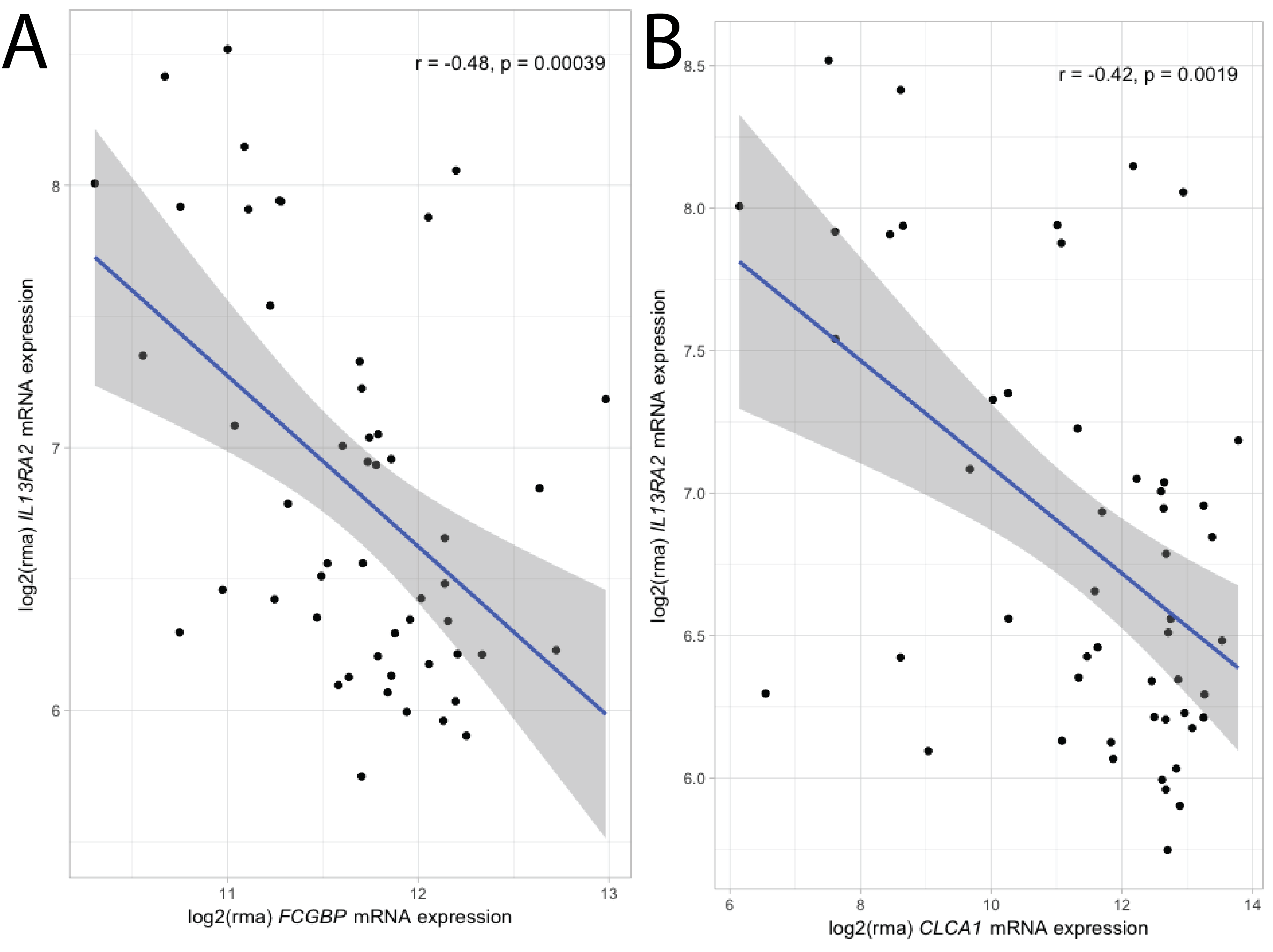

Supplement: Supplementary Figure 1 — Correlation between IL13RA2 mRNA and goblet specific cell genes. Correlations between expression of IL13RA2 with the expression of FCGBP (A) and CLCA1 (B), backbone of the colonic mucus layer. [file Image_1.TIFF]
